# Mechano Growth Factor Accelerates ACL Repair and Improves Cell Mobility of Mechanically Injured Human ACL Fibroblasts by Targeting Rac1-PAK1/2 and RhoA-ROCK1 Pathways

**DOI:** 10.3390/ijms23084331

**Published:** 2022-04-14

**Authors:** Yongqiang Sha, Beibei Zhang, Liping Chen, Huhai Hong, Qingjia Chi

**Affiliations:** 1 Center for Precision Medicine, School of Medicine and School of Biomedical Sciences, Huaqiao University, Xiamen 361021, China; zhangbeibei2299@163.com (B.Z.); ch990721@163.com (L.C.); h18359890781@163.com (H.H.); 2 National Innovation and Attracting Talents “111” Base, Key Laboratory of Biorheological Science and Technology, Ministry of Education, College of Bioengineering, Chongqing University, Chongqing 400030, China; qingjia@whut.edu.cn; 3 Department of Mechanics and Engineering Structure, Hubei Key Laboratory of Theory and Application of Advanced Materials Mechanics, Wuhan University of Technology, Wuhan 430070, China

**Keywords:** mechano growth factor, anterior cruciate ligament, cell migration, cell adhesion, fracture strength

## Abstract

Exceeded mechanical stress leads to a sublethal injury to anterior cruciate ligament (ACL) fibroblasts, and it will hinder cell mobility and ACL regeneration, and even induce osteoarthritis. The mechano growth factor (MGF) could be responsible for mechanical stress and weakening its negative effects on cell physiological behaviors. In this study, effects of MGF on cell mobility and relevant molecules expression in injured ACL fibroblasts were detected. After an injurious mechanical stretch, the analysis carried out, at 0 and 24 h, respectively, showed that the cell area, roundness, migration, and adhesion of ACL fibroblasts were reduced. MGF (10, 100 ng/mL) treatment could improve cell area, roundness and promote cell migration and adhesion capacity compared with the injured group without MGF. Further study indicated that cell mobility-relevant molecules (PAK1/2, Cdc42, Rac1, RhoA, and ROCK1) expression in ACL fibroblasts was down-regulated at 0 or 24 h after injurious stretch (except Rac1 and RhoA at 0 h). Similarly, MGF improved cell mobility-relevant molecule expression, especially the ROCK1 expression level in ACL fibroblasts at 0 or 24 h after injurious stretch. Protein expression of ROCK1 in injured ACL fibroblasts was also reduced and could be recovered by MGF treatment. In a rabbit partial ACL transection (ACLT) model, ACL exhibited poor regenerative capacity in collagen and extracellular matrix (ECM) synthesis after partial ACLT for 2 or 4 weeks, and MGF remarkably accelerated ACL regeneration and restored its mechanical loading capacity after partial ACLT for four weeks. Our findings suggest that MGF weakens the effects of pathological stress on cell mobility of ACL fibroblasts and accelerates ACL repair, and might be applied as a future treatment approach to ACL rupture in the clinic.

## 1. Introduction

The anterior cruciate ligament (ACL) is the major mechanical loading tissue in the human knee joint, maintaining the stability and biomechanical properties of the joint. The prevalence of ACL injury has increased due to intense sport activities and inappropriate mechanical load, whereas it is hard for ACL to self-heal [1,2]. The response being deficient in growth factors [3], severe hypoxic condition [4], inflammatory reaction [5], lacking blood supply [6], and other factors restrict timely ACL regeneration after tissue injury. Deficiency of cell proliferation [7] and mobility [8] capacity also hinder ACL regeneration. In addition, previous studies confirmed that an abnormal mechanical environment would induce a chronic ACL repair process and weaken the final mechanical properties after ACL repair [9]. However, whether abnormal mechanical stress will threaten cell mobility of ACL fibroblasts is not known and needs to be explored.

During daily sports activities, sudden rotation and landing will change the mechanical loading on ACL and raise the prevalence of ACL injury [10]. Moreover, mechanical loading distribution on ACL is not uniform and concentrates on lateral fiber bundles from the middle part of the ligament to the femoral insertion, which are more easily torn [11,12]. ACL injuries most often happen late in the day and season, or at a later stage of sports, suggesting that fatigue plays a role [13,14]. In other words, although intense or long-term sports may not induce ACL injury, they will cause cell damage to the ACL fibroblasts, maintaining and elevating the incidence of ACL injuries [14]. To improve or restore cell properties of fatigue damaged ACL fibroblasts may be helpful for preventing or reducing the risk of ACL injury.

Using growth factors for tissue repair and treatment was reported to be effective in the previous studies. The mechano growth factor (MGF) is the splice variant of the insulin-like growth factor 1 (IGF-1) in an autocrine or paracrine manner [15,16], and can be up-regulated in osteoblasts [17], cardiomyocytes [18], or neurons [19] withstanding mechanical stress or hypoxia-ischemia, protecting these cells from injury and accelerating tissue regeneration. The functional E domain of MGF (MGF E peptide) is confirmed to have a similar function, with the full-length MGF accelerating tissue repair and retaining cell physiology under different disadvantageous environments. A number of studies reported that MGF could protect BMSCs, chondrocytes, and ACL fibroblasts from severe hypoxia-induced damage [20,21,22]. Additionally, MGF exhibited excellent effects in protecting cardiomyocytes or neurons from severe hypoxia-induced cell apoptosis [18,19]. Furthermore, MGF also showed an effectively protective role in avoiding mechanical stimulation induced damage. MGF was previously reported to play a critical role in protecting tissues from exceeded mechanical stress [23,24] and stimulating muscle and neuronal repair [16]. Our previous studies confirmed that pretreatment with MGF could effectively keep mesenchymal stem cells (MSCs) from high-intensity fluid shear stress (FSS)-induced negative effects, such as obvious reduction of cell viability and proliferation of MSCs [25]. Moreover, MGF also promoted cell viability and proliferation of ACL fibroblasts withstanding injurious stretch. That MGF could enhance various kinds of cells or tissues so that they resist injured stress, whether or not it also has the favorable utility of recovering cell mobility of injured ACL fibroblasts, should be detected exhaustively.

**Figure 1:**
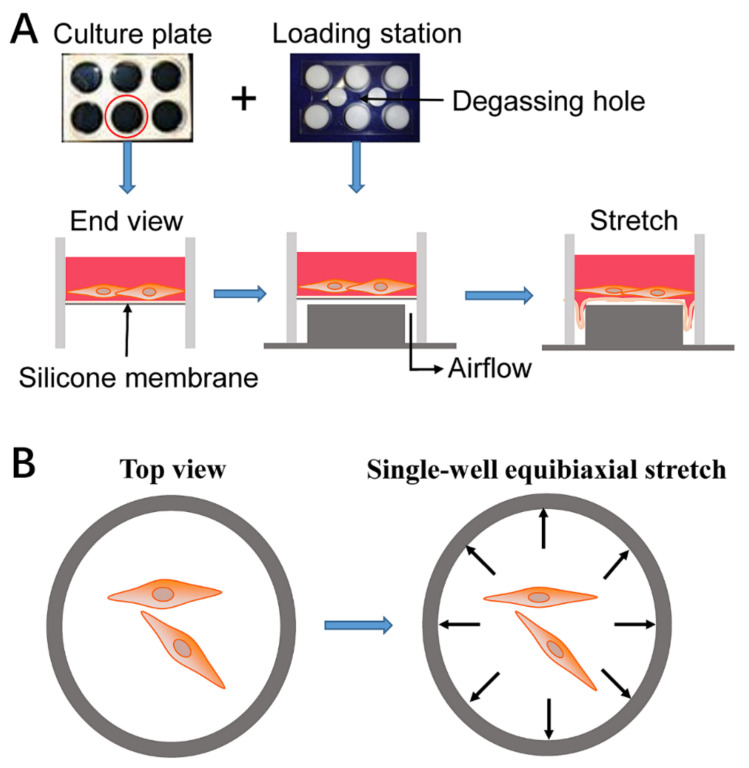
Schematic presentation of the experimental procedure. (**A**) Schematic presentation of the mechanical stretch performed by six-well flexible silicone rubber BioFlex^TM^ plates coated with collagen I. (**B**) Schematic diagram of equi-biaxial tension.

In the current study, 12% equi-biaxial static stretch was performed on human ACL fibroblasts to simulate the injured stimulation. Mechanical stretch was performed as shown in Figure 1. Furthermore, MGF was used to detect its effects on cell morphology, migration, adhesion, and relevant molecule expression levels of human ACL fibroblasts withstanding 12% static injurious stretch. In addition, rabbit partial ACL transection (ACLT) surgery was carried out, and whether MGF could accelerate ACL repair and restore its mechanical loading capacity after partial ACLT for 2 or 4 weeks was also verified.

## 2. Results

### 2.1. MGF Improved Cell Deformation of ACL Fibroblasts after Injurious Stretch

**Figure 2:**
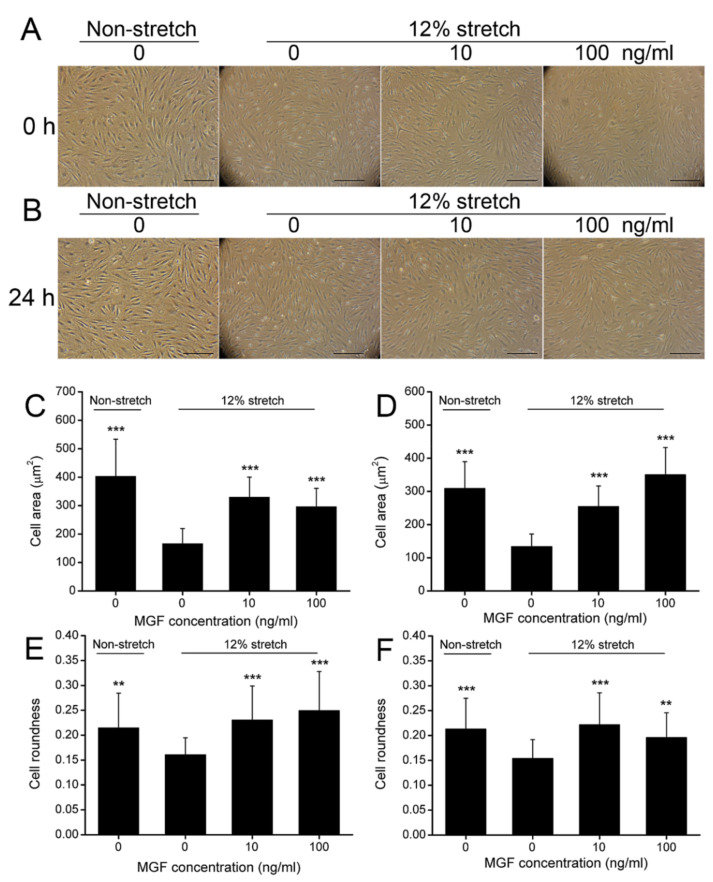
Mechano growth factor (MGF) improved cell morphology of human anterior cruciate ligament (ACL) fibroblasts withstanding injured stretch. Cell morphological changes in human ACL fibroblasts in non-stretch, 12% stretch, (10, 100 ng/mL) MGF + 12% stretch groups at (**A**) 0 or (**B**) 24 h after injurious stretch. The quantitative results of cell area detected at (**C**) 0 or (**D**) 24 h. The quantitative results of cell roundness detected at (**E**) 0 or (**F**) 24 h. Scar bar = 100 μm. Data are presented as mean ± SD. **, *p* < 0.01; ***, *p* < 0.001 compared to the control group (the 12% stretch without MGF group).

ACL fibroblasts were isolated and identified through observing cell morphology and detecting the relevant cell markers. CD31^−^/CD34^+^/CD44^+^/Collagen I^+^/Collagen III^+^/α-SMA^+^ cells were considered to be ACL fibroblasts (Figure S1). Subsequently, cell area and roundness were verified to represent cell deformability of ACL fibroblasts at 0 or 24 h after injurious stretch (Figure 2A,B). The results showed that cell area was reduced from 403.35 ± 130.27 μm^2^ (*p* < 0.001) to 166.30 ± 53.16 μm^2^ at 0 h (*p* < 0.001), and 308.69 ± 80.74 μm^2^ to 133.15 ± 38.57 μm^2^ at 24 h (*p* < 0.001) (Figure 2C,D). MGF could recover the cell area of ACL fibroblasts reduced by the injurious stretch. It was found that 10 ng/mL MGF increased cell area of injured ACL fibroblasts to 329.65 ± 70.47 μm^2^ (*p* < 0.001) at 0 h, 254.25 ± 62.04 μm^2^ (*p* < 0.001) at 24 h after stretch (Figure 2C,D). Moreover, 100 ng/mL MGF was also beneficial to cell deformation, and increased the cell area of the injured ACL fibroblasts to 286.66 ± 64.08 μm^2^ (*p* < 0.001) at 0 h, 349.78 ± 82.64 μm^2^ (*p* < 0.001) at 24 h after stretch (Figure 2C,D). Cell roundness was also regulated by MGF, and it was found that cell roundness of injured ACL fibroblasts decreased from 0.2147 ± 0.0699 to 0.1607 ± 0.0342 at 0 h (*p* < 0.01), and 0.2128 ± 0.0619 to 0.1539 ± 0.0378 (*p* < 0.001) at 24 h as well (Figure 2E,F). Simultaneously, 10 ng/mL MGF raised cell roundness of injured ACL fibroblasts to 0.2302 ± 0.0685 at 0 h (*p* < 0.001) and 0.2215 ± 0.0642 at 0 h (*p* < 0.001) (Figure 2E,F). In addition, 100 ng/mL MGF raised cell roundness of injured ACL fibroblasts to 0.2495 ± 0.0785 at 0 h (*p* < 0.001) and 0.1958 ± 0.0497 at 0 h (*p* < 0.001) (Figure 2E,F).

### 2.2. MGF Improved Cell Migration of ACL Fibroblasts after Injurious Stretch

**Figure 3:**
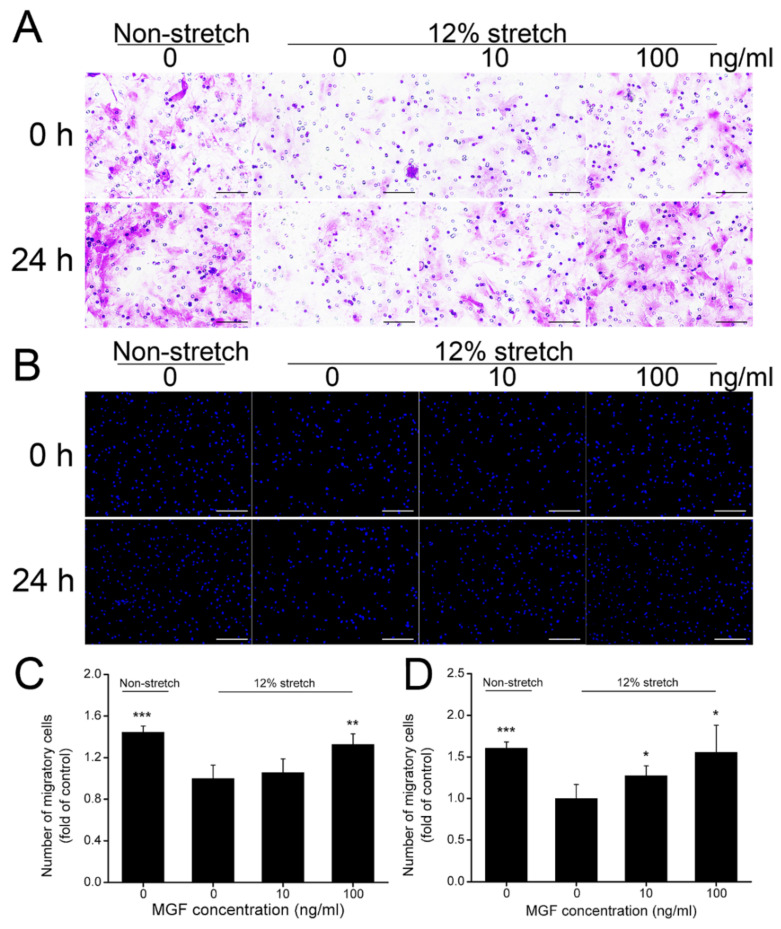
Mechano growth factor (MGF) promoted cell migration of human anterior cruciate ligament (ACL) fibroblasts withstanding injurious stretch. Effects of MGF on cell migration of human ACL fibroblasts were detected through (**A**) crystal violet and (**B**) DAPI staining at 0 or 24 h after injurious stretch. Quantitative results of migratory cells of human ACL fibroblasts at (**C**) 0 or (**D**) 24 h after injurious stretch. Scale bar = 200 μm. Data are presented as mean ± SD. *, *p* < 0.05; **, *p* < 0.01; ***, *p* < 0.001 compared to the 12% stretch group without MGF.

The number of ACL fibroblasts migrating to the injured site play a key role in accelerating ACL repair after tearing damage. In the current study, cell migration of ACL fibroblasts was verified through crystal violet and DAPI staining at 0 or 24 h after injurious stretch separately (Figure 3A,B). Statistical results indicated that injurious stretch reduced cell migratory rate from 144.38 ± 5.91% to 100.00 ± 12.78% (*p* < 0.001) compared to the non-stretch group at 0 h, and 10 and 100 ng/mL MGF improved cell migratory rate to 105.62 ± 13.23% (*p* = 0.56) and 132.58 ± 10.22% (*p* < 0.01) compared to the 12% stretch without MGF group (the control group) separately (Figure 3C). After continuous incubation for 24 h, cell migratory rate was reduced from 160.44 ± 7.61% to 100.00 ± 16.88% (*p* < 0.001) compared to the non-stretch group, and 10 and 100 ng/mL MGF improved cell migratory rate to 127.47 ± 11.90% (*p* < 0.05) and 155.49 ± 32.72% (*p* < 0.05) compared to the 12% injurious stretch without MGF group separately (Figure 3D).

### 2.3. MGF Increased Cell Adhesion of ACL Fibroblasts after Injurious Stretch

**Figure 4:**
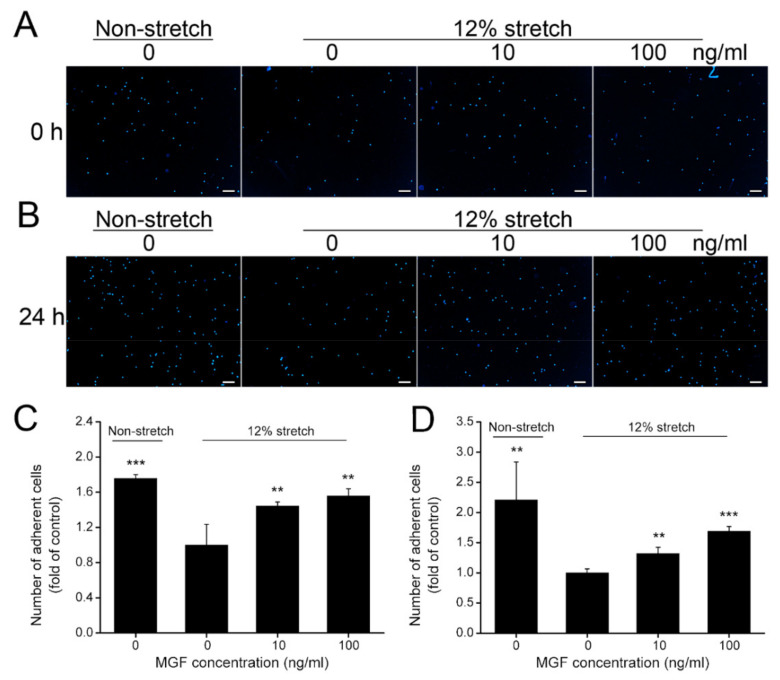
Mechano growth factor (MGF) accelerated cell adhesion of human anterior cruciate ligament (ACL) fibroblasts withstanding injurious stretch. Cell adhesion of human ACL fibroblasts was detected using DAPI staining at (**A**) 0 or (**B**) 24 h after injurious stretch. Quantitative results of adherent cells of human ACL fibroblasts at (**C**) 0 or (**D**) 24 h after injurious stretch. Scale bar = 100 μm. Data are presented as mean ± SD. **, *p* < 0.01; ***, *p* < 0.001 compared to the 12% stretch without MGF group.

Cell adherent capacity is a critical factor in regulating cell mobility and it is meaningful for tissue repair. As shown in Figure 4, adherent ACL fibroblasts stained with DAPI were reduced from 175.65 ± 4.49% to 100.00 ± 23.48% (*p* < 0.001) at 0 h after injurious stretch, and 10, 100 ng/mL MGF increased cell adherent rate to 144.35 ± 4.49% (*p* < 0.01) and 155.65 ± 8.22% (*p* < 0.01) compared to the control group (Figure 4A,C). Moreover, cell adhesion of ACL fibroblasts was also decreased from 220.87 ± 62.82% to 100.00 ± 6.585 at 24 h (*p* < 0.01), 10, 100 ng/mL MGF increased cell adherent rate to 132.17 ± 10.24% (*p* < 0.01) and 169.13 ± 7.70% (*p* < 0.001) compared to the control group (12% stretch without MGF group) (Figure 4B,D).

### 2.4. MGF Regulated Cell Mobility-Relevant Molecules Expression in ACL Fibroblasts after Injurious Stretch

**Figure 5:**
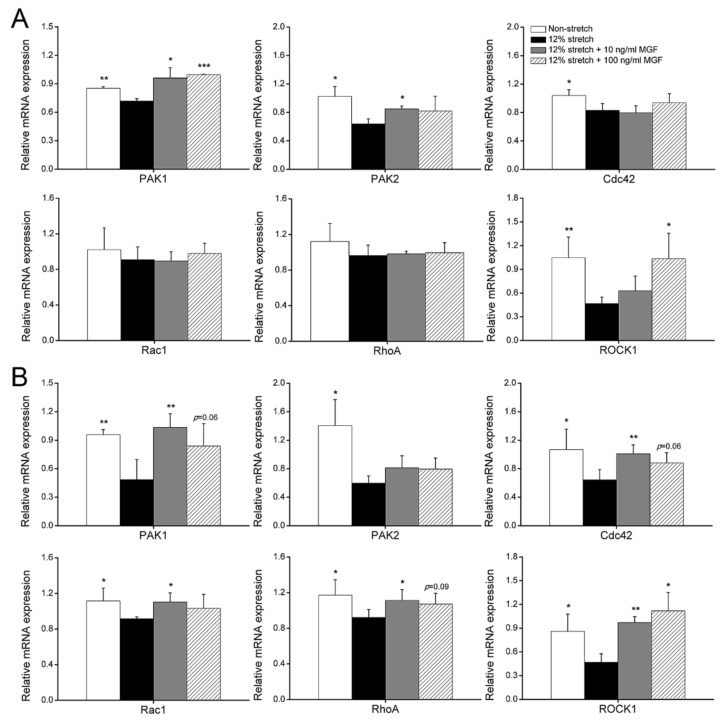
Mechano growth factor (MGF) regulated mRNA expression levels of cell motility-relevant genes in injured human anterior cruciate ligament (ACL) fibroblasts. Effects of MGF on mRNA expression of PAK1, PAK2, Cdc42, Rac1, RhoA, and ROCK1 in human ACL fibroblasts at (**A**) 0 or (**B**) 24 h after injurious stretch. Data are presented as mean ± SD. *, *p* < 0.05; **, *p* < 0.01; ***, *p* < 0.001 compared to the 12% stretch group without MGF.

Small GTPases of ras homologue family (Rho) and P21-activated kinase 1/2 (PAK1/2) are the typical cell morphology and mobility-relevant molecules and detected in this study. It was found that mRNA expression levels of PAK1, PAK2, Cdc42, and ROCK1 were reduced by 15.75% (*p* < 0.01), 37.72% (*p* < 0.05), 20.09% (*p* < 0.05), and 55.39% (*p* < 0.001) in ACL fibroblasts at 0 h after injurious stretch, and 10 ng/mL MGF increased PAK1 and PAK2 mRNA expression levels from 0.7191 ± 0.0251 to 0.9623 ± 0.1075 (*p* < 0.05), and from 0.6387 ± 0.0703 to 0.8498 ± 0.0398 (*p* < 0.05), but 100 ng/mL MGF only improved PAK1 and ROCK1 mRNA expression levels and increased them to 0.9973 ± 0.0047 (*p* < 0.001), 1.0348 ± 0.3204 (*p* < 0.05) (Figure 5A). Furthermore, mRNA expression of PAK1, PAK2, Cdc42, Rac1, RhoA, and ROCK1 were reduced in injured ACL fibroblasts by 49.43% (*p* < 0.01), 57.56% (*p* < 0.05), 39.72% (*p* < 0.05), 17.83% (*p* < 0.05), 21.38% (*p* < 0.05), and 45.50% (*p* < 0.05) separately after continuous incubation for 24 h (Figure 5B). Furthermore, 10 ng/mL MGF obviously increased mRNA expression levels of PAK1, Cdc42, Rac1, RhoA and ROCK1 from 0.485 ± 0.2108 to 1.0343 ± 0.1449 (*p* < 0.01), from 0.6443 ± 0.1447 to 1.0104 ± 0.125 (*p* < 0.01), from 0.9162 ± 0.0213 to 1.1035 ± 0.1034 (*p* < 0.05), from 0.921 ± 0.0904 to 1.1137 ± 0.1219 (*p* < 0.05) and from 0.4682 ± 0.1076 to 0.9705 ± 0.0747 (*p* < 0.01) compared with the 12% stretch without MGF group, whereas 100 ng/mL MGF improved PAK1, Cdc42, RhoA, and ROCK1 mRNA expression to 0.8396 ± 0.2342 (*p* = 0.06), 0.8796 ± 0.1454 (*p* = 0.06), 1.0709 ± 0.1225 (*p* = 0.09) and 1.1183 ± 0.2321 (*p* < 0.05), respectively (Figure 5B).

### 2.5. MGF Regulated ROCK1 Protein Expression in ACL Fibroblasts after Injurious Stretch

**Figure 6:**
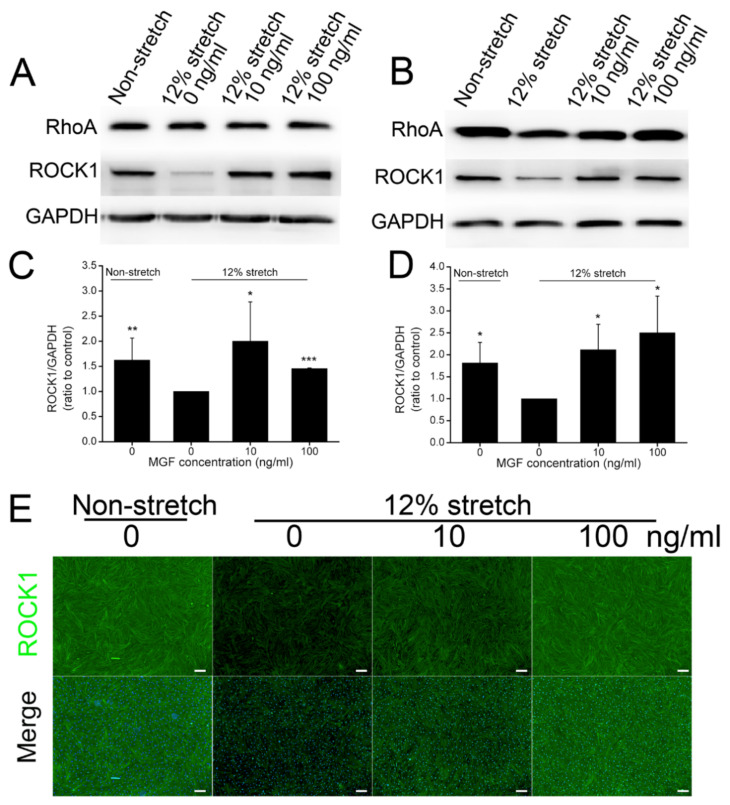
Mechano growth factor (MGF) increased ROCK1 protein expression level in human anterior cruciate ligament (ACL) fibroblasts withstanding injured stretch. (**A**,**B**) Western blotting analysis and (**C**,**D**) quantified ROCK1 protein expression levels in human ACL fibroblasts in non-stretch, 12% stretch, (10, 100 ng/mL) MGF + 12% stretch groups at 0 or 24 h after injurious stretch. (**E**) ROCK1 protein expression levels in human ACL fibroblasts in abovementioned four groups at 24 h after injurious stretch. Scale bar = 200 μm. Data are presented as mean ± SD. *, *p* < 0.05; **, *p* < 0.01; ***, *p* < 0.001 compared to the 12% stretch group without MGF.

A number of previous studies confirmed that ROCK1 kept a tight touch with cell mobility and adhesion [26,27]. Protein expression levels of ROCK1 were also analyzed using Western blotting and immunofluorescence analysis (Figure 6). The results showed that ROCK1 expression decreased by 38.43% (*p* < 0.01) in injured ACL fibroblasts, and 10, 100 ng/mL MGF could recover its expression by 100.19 ± 78.17% (*p* < 0.05) and 45.65 ± 1.06% (*p* < 0.001) separately compared with the 12% injurious stretch without MGF group at 0 h (Figure 6A,C). After continuous incubation for 24 h, 12% injurious stretch reduced ROCK1 protein expression by 44.87% (*p* < 0.05), whereas 10 and 100 ng/mL MGF increased ROCK1 expression by 111.44 ± 57.91% (*p* < 0.05) and 150.17 ± 83.52% (*p* < 0.05) separately (Figure 6B,D). Moreover, immunofluorescence staining exhibited a similar result with Western blotting, and MGF could also weaken the negative effects of injurious stretch on ROCK1 expression in ACL fibroblasts at 24 h (Figure 6E).

### 2.6. MGF Accelerated ACL Regeneration after Partial ACLT Surgery

**Figure 7:**
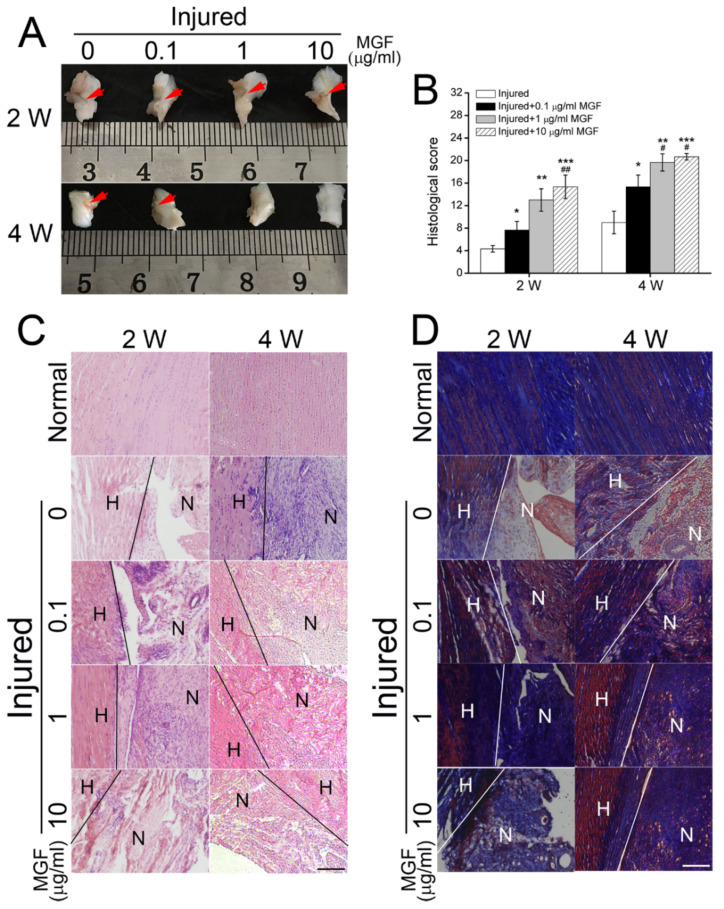
Mechano growth factor (MGF) accelerated ACL regeneration through up-regulating extracellular matrix synthesis after anterior cruciate ligament (ACL) transection surgery in vivo. (**A**) Macroscopically evaluation of ACL in the ACLT (the control group) and (0.1, 1 and 10 μg/mL) MGF + ACLT groups after ACL partial transection for 2 or 4 weeks. Red arrows: ACL transection sites. (**B**) Histological scores. (**C**) Hematoxylin-Eosin (HE) and (**D**) Masson staining of ACL tissue after ACL injured surgery for 2 or 4 weeks. H: host tissue; N: new-born tissue. Scale bar = 100 μm. Data are presented as mean ± SD. *, *p* < 0.05; **, *p* < 0.01; ***, *p* < 0.001 compared to the ACLT group without MGF. #, *p* < 0.05; ##, *p* < 0.01 compared to the ACLT group with 0.1 μg/mL MGF.

Macroscopic analysis found lots of temporary extracellular matrix (ECM) filled in the injured sites after ACL transection (ACLT) surgery, subsequently kinds of cells would migrate along the ECM and began to expansion for followed tissue regeneration. Histological score assay indicated that MGF accelerated ACL repair after injured surgery for 2 and 4 weeks (Figure 7A,B). After partial ACLT for 2 weeks, 0.1, 1 and 10 μg/mL MGF increased the histological score of ACL tissue maturity from 4.3333 ± 0.5774 to 7.6667 ± 1.5275 (*p* < 0.05), 13.0000 ± 2.0000 (*p* < 0.01) and 15.3333 ± 2.0817 (*p* < 0.001) compared to the injured group (Figure 7A,B). Moreover, histological score in 10 μg/mL MGF group showed better appearance than the 0.1 μg/mL MGF group. After 4 weeks, 0.1, 1 and 10 μg/mL MGF increased the histological score of ACL tissue maturity from 9.0000 ± 2.0000 to 15.3333 ± 2.0817 (*p* < 0.05), 19.6667 ± 1.5275 (*p* < 0.01) and 20.6667 ± 0.5714 (*p* < 0.001) compared with the injured group (Figure 7A,B). Furthermore, both 1 μg/mL and 10 μg/mL MGF groups showed better appearance than the 0.1 μg/mL MGF group.

### 2.7. MGF Improved ECM Synthesis of ACL after Partial ACLT Surgery

Hematoxylin-Eosin (HE) and Masson staining represented collagen and other ECM synthesis, and even inflammatory reaction. HE staining assay showed that few fibroblasts and inflammatory cells migrated to the injured sites in the injured groups, and ECM synthesis was less. However, 0.1, 1 and 10 μg/mL MGF groups promoted more fibroblasts and inflammatory cells migrating to the injured sites than the injured group after partial ACLT for 2 weeks, and number of inflammatory cells in 10 μg/mL MGF group was less than 0.1 and 1 μg/mL MGF groups (Figure 7C). After partial ACLT for 4 weeks, many inflammatory cells migrated to the injured sites in the injured group, and only a few inflammatory cells but a number of fibroblasts existed in MGF treatment groups, and obvious bundles parallel to the long axis of ligament (Figure 7C). Masson staining assay indicated that MGF treatment could significantly promote collagen synthesis after partial ACLT for 2 or 4 weeks (Figure 7D), which is beneficial to ACL repair.

### 2.8. MGF Improved Mechanical Properties of ACL after Partial ACLT Surgery

**Figure 8:**
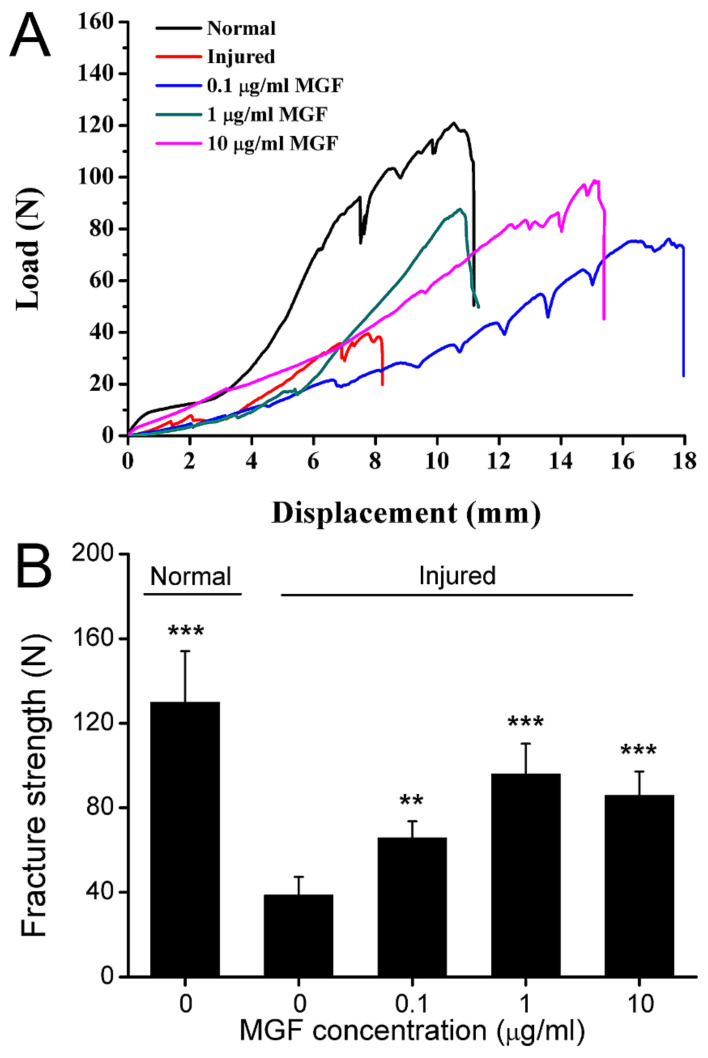
Mechano growth factor (MGF) restored the mechanical capacity of anterior cruciate ligament (ACL) after partial transection surgery. (**A**) Stress-strain curve of ACL in the normal, ACLT and (0.1, 1 and 10 μg/mL) MGF + ACLT groups after ACL transection for 4 weeks. (**B**) Quantitative results of the largest fracture strength in abovementioned groups. Data are presented as mean ± SD. **, *p* < 0.01; ***, *p* < 0.001 compared to the ACLT group without MGF.

After partial ACLT for four weeks, mechanical properties of ACL in normal, injured, 0.1 μg/mL MGF, 1 μg/mL MGF and 10 μg/mL MGF groups were detected stress-strain assay and largest fracture strength was counted. It was shown that MGF treatment obviously promoted the mechanical properties of ACL compared with the injured group, and this was similar to normal ACL (Figure 8A). In addition, fracture strength in the injured group was still remarkably lower than normal ligament. The results showed that the fracture strength was reduced from 129.92 ± 24.16 N to 38.60 ± 8.72 N (*p* < 0.001) in the injured group, and 0.1, 1 and 10 μg/mL MGF increased the fracture strength from 38.60 ± 8.72 N to 65.71 ± 7.85 N (*p* < 0.01), 95.98 ± 14.39 N (*p* < 0.001), 85.75 ± 11.40 N (*p* < 0.001) separately (Figure 8B).

## 3. Discussion

Favorable cell morphology [28,29], migration capacity [30], and adhesion force [31] were beneficial for ACL regeneration and functional remodeling after ACL injury or reconstruction, avoiding mechanical destabilization [32], more serious meniscus degeneration [33], and articular cartilage degradation [34]. In the current study, human ACL fibroblasts were harvested, seeded on the six-well flexible silicone rubber BioFlex^TM^ plates coated with collagen I, and followed subjected to the 12% injurious stretch using FX-4000T^TM^ Flexcell^®^ Tension Plus^TM^ unit. Subsequently, effects of injurious mechanical stretch and MGF on cell area, roundness, migration, adhesion, and cell deformation-relevant molecules in ACL fibroblasts were detected. The results indicated that cell area and roundness of ACL fibroblasts were significantly reduced. Cytoplasm in injured ACL fibroblasts was also reduced and cell polarity became more distinct. MGF treatment could protect ACL fibroblasts from the injurious stretch-induced decline of cellular morphological recovery capacity. Moreover, Cell migration and adhesion capacity of injured ACL fibroblasts were also obviously decreased compared with normal cells, and MGF weakened the negative effects caused by injurious stretch on cell migration and adhesion of ACL fibroblasts. Rac1/Cdc42/PAK1/PAK2 and RhoA/ROCK1 pathways might be involved in MGF regulating cell morphology, migration and adhesion of ACL fibroblasts withstanding injurious mechanical stretch. In rabbit partial ACLT models, MGF shorten the inflammatory phase and recruited types of cells migrating to the injured sites for the followed ACL repair. Abundant collagen fibers and other ECM were synthesized. In addition, MGF was confirmed to be effective in recovering the mechanical properties of ACL. In the completely ACLT rabbit models, MGF exhibited an excellent effects on attenuating osteoarthritis after 2 months (Figure S3).

Mechanical stimulation is an important regulatory factor involved in a number of cell behaviors. Physiological mechanical conditions always stimulate cell proliferation of synovial cells [35], viability, and differentiation of mesenchymal stem cells (MSCs) [36], whereas exceeded mechanical stretch exhibits negative effects on cell behaviors. Previous studies confirmed that injurious stretch-induced metalloproteinase 2/9 (MMP 2/9) expression and activity in some types of cells [37,38]. Our study confirmed that injured static stretch confused the ECM metabolism and reduced cell proliferation of human ACL fibroblasts [39]. In addition, ACL injury induced by exceeded mechanical stress could not restore itself, but no cell apoptosis occurred, suggesting the ACL fibroblasts with sublethal injury remained in the ligament and restricted ACL regeneration [39]. In addition, an accumulation of the injured ACL fibroblasts through daily intensive exercise will result in a higher prevalence of ACL rupture.

After ACL rupture partly, the repair process consists of four histological phases, i.e., inflammation, epiligamentous regeneration, proliferation, and the remodeling phase [40]. Neutrophils and macrophages were recruited to the injured sites in the inflammation phase to retrieve the ACL remnant, to supply a better basis for ACL repair [41,42]. Subsequently, temporary ECM composed of hyaluronic acid forms soon after ACL injury, and massive ACL fibroblasts migrate into the temporary ECM, expanding and producing collagen and other ECM [40]. However, a prolonged inflammatory phase is detrimental to the follow-up tissue repair [43]. Early termination of inflammatory response is critical for a nice ACL repair effect. As shown in Figure 7C, MGF could advance and complete the inflammatory response, and accelerate ACL repair progression.

Once ACL rupture completely, autologous tissues transplant is considered to be the ‘golden standard’ for ACL reconstruction, including parts of patellar ligament with its bony patellar and tibial attachments or hamstring tendons [44]. Due to the limitation of autologous tissue source and ‘donor site morbidity’ [44], suitable biomaterial-based transplant has been frequently used instead of autologous tissues [45,46]. Previous clinical results confirmed that remaining ACL remnants are essential for its repair and mechanical properties after ACL single- or double-bundle reconstruction surgery [47]. Guided tissue regeneration of the ruptured ACL offers potential benefits for retaining the complex footprints of the ACL and the proprioceptive nerve fibers [48]. However, ACL remnant-derived cells migrating to the adjacent regeneration template or scaffold after rupture is the fundamental process for ACL reconstruction and guarantees the reconstruction to be successful [9,48].

Cell adhesion capacity is another critical factor for ACL reconstruction and functional recovery. During the ACL reconstruction process, periostin will be highly expressed in the ACL remnant-derived progenitor cells to increase cell adhesion potential [49]. Remnant tissue-derived progenitor cells can adhere to the bony tunnel and create a lower femoral tunnel, preventing ACL mechanical destabilization again [50]. Moreover, for a functionalized and suitable ACL transplant, it should allow the ACL fibroblasts’ adhesion, migration, and promote the production of ECM to build up a new ACL [51], i.e., positive cell mobility is beneficial to not only ACL regeneration itself but ACL reconstruction with types of transplants.

## 4. Materials and Methods

### 4.1. Cell Isolation and Culture

ACL fibroblasts used in the experiment were extracted from four donors (2 males and 2 females) who accepted total knee replacement surgery. The operations were performed at the First Affiliated Hospital (Chongqing, China) and the ACL fibroblasts were extracted according to the previous study [20]. All the experimental processes were performed according to ethical principles and protocols approved by Huaqiao University. Patient consent was gained before the knee replacement surgery. All the donors were healthy and had no inflammatory response in the knee articular cavity, i.e., the patients did not suffer from osteoarthritis, rheumatoid arthritis, or any long-term pathological changes in the knee articular cavity, so that the ACL tissue-derived fibroblasts were ensured to be normal. All the ACL tissues were immediately infiltrated into the sterile phosphate-buffered saline (PBS) with 5× penicillin and 5× streptomycin sulfate once harvested. Subsequently, the tissues were cut into small pieces. These tissues pieces were moved into Dulbecco’s modified eagle medium (DMEM) (Gibco, Darmstadt, Germany) containing 10% fetal bovine serum (FBS) (Gibco, Darmstadt, Germany). Then, the flasks were put in a humidified atmosphere to guarantee cell growth. Once ACL fibroblasts migrated from the tissues and grew, small pieces were moved to a new flask. When the ACL fibroblasts began to converge, the cells were trypsined and divided into three new flasks. Cells extracted from different donors were stored as a separate sample. All the procedures were performed at least four times in the current study.

### 4.2. Cell Stretch

ACL fibroblasts were planted in the silicone rubber membrane coating collagen I at a density of 1 × 10^4^ cells/cm^2^ in DMEM supplemented with 10% FBS. After continuous culture for 24 h, the medium was removed and 2 mL fresh DMEM supplemented with 2% FBS was added to starve cells. After cell starvation, ACL fibroblasts were pretreated with MGF (Beyotime, Beijing, China) (0, 10 or 100 ng/mL) in DMEM with 1% FBS for 24 h. Afterwards, the media was removed and ACL fibroblasts were washed using sterile PBS three times. Then, 2 mL fresh DMEM with 1% FBS was added and all samples were exposed to 12% static stretch (injurious stretch) for 6 h using the FX-4000T^TM^ Flexcell^®^ Tension Plus^TM^ unit (Flexcell International Corporation, Burlington, NC, USA) [52,53]. Before the mechanical stretch experiment, the stretching range (12%), frequency, time, and load model were set using the software, and the equipment would automatically calculate the required vertical displacement of the rubber membrane, which could ensure that cell length was elongated 12% in the circumferential direction. Thus, four groups (non-stretch group, 12% stretch without MGF group, 12% stretch with 10 ng/mL MGF group, 12% stretch with 100 ng/mL MGF group) were set. In addition, 12% stretch without MGF group was considered to be the control group in this study. Subsequently, the ACL fibroblasts were continuously incubated after 12% injurious stretch. To analyze the potential effects induced through 12% injurious mechanical stress and whether they could be restored, cell morphology, migration, adhesion, cell mobility-relevant molecules expression were detected at 0 or 24 h. Finally, effects of MGF on ACL regeneration were further verified.

### 4.3. Cell Area and Roundness

Cell deformation of ACL fibroblasts after injurious stretch was represented through analyzing cell area and roundness. After 12% injurious stretch of human ACL fibroblasts, the cells were photographed by fluorescence microcopy at 0 and 24 h, respectively (Olympus, Tokyo, Japan). Cell area and roundness were verified by Image J software (1.46r, NIH Image J Software, Bethesda, MD, USA). Briefly, the pictures of ACL fibroblasts morphology were opened, and the accurate scale bar must be drawn. Then, polygon tools were used, and cell edge was depicted as accurately as possible. Finally, measurement was chosen, and details of cell area and roundness were exhibited in following pop-up table.

### 4.4. Cell Migration

After 12% injurious stretch of human ACL fibroblasts, cell migration was verified by transwell and DAPI staining analysis at 0 or 24 h. Briefly, 50 μL matrigel matrix (BD Corp, Franklin Lakes, NJ, USA) was added to the tranwell inserts (Milliopore, Burlington, MA, USA) with polycarbonate membranes with 8-μm pores and dried in the incubator at 37 °C for 4 h. Subsequently, 50 μL DMEM without serum was added to the inserts. At 0 or 24 h after injurious stretch, ACL fibroblasts were trypsinized, centrifuged at 300 g × 5 min and resuspended with the serum-free medium. Then, approximately 6 × 10^4^ ACL fibroblasts in 150 μL medium were seeded into the inserts and the inserts were transferred in the 24-well plates with 600 μL L-DMEM + 10% FBS for continuous culture for 48 h. Then, the inner surfaces of the inserts were wiped slightly to clean the remaining ACL fibroblasts. Finally, cell migration was analyzed using 0.05% crystal violet and DAPI staining and were photographed with fluorescence microscopy (Olympus, Tokyo, Japan).

### 4.5. Cell Adhesion

After 12% injurious stretch of human ACL fibroblasts, cell adherent capacity was verified at 0 or 24 h. The cells were washed using sterile PBS and trypsinized. The cells were collected and centrifuged. Subsequently, ACL fibroblasts were resuspended using 1% FBS fresh DMEM. Then, approximately 5 × 10^4^ cells in each sample were planted into the 24-well plates. The ACL fibroblasts began to attach to the plate. After 30 min, the ACL fibroblasts were washed using sterile PBS three times to abandon the still suspended cells. The adherent ACL fibroblasts were analyzed using DAPI staining and examined using fluorescence microscopy.

### 4.6. Quantitative Real-Time Polymerase Chain Reaction

After 12% injurious stretch of human ACL fibroblasts, expression levels of cell deformation-relevant genes were verified at 0 or 24 h. Expression of cell cytoskeleton arrangement- and mobility-relevant genes was examined by qRT-PCR. The RNA sample was extracted using RNAsimple total RNA kit (DP419; TIANGEN, Beijing, China) and quantified by measuring the absorbance at 260 nm. Followed RNA samples were reverse-transcribed into complementary DNA (cDNA) using RevertAid^TM^ First Strand cDNA synthesis Kit (Thermo, Waltham, MA, USA) according to the manufacturer’s instruction. The primers of Cdc42, Rac1, RhoA, ROCK1, PAK1, PAK2 and GAPDH are shown in Table 1. Specificity and amplification efficiency of the primers were analyzed by the melt curve and Ct values (Figure S3). The experiments were performed in Bio-Rad CFX96 Real-Time PCR system. Cdc42, Rac1, RhoA, ROCK1, PAK1, PAK2 were carried out with the same cDNA sample and GAPDH was set as the internal control.

### 4.7. Western Blotting Assay

After 12% injurious stretch of human ACL fibroblasts, expression levels of cell deformation-relevant proteins were verified at 0 or 24 h. Protein expression of RhoA, ROCK1 and GAPDH were detected to present cell cytoskeleton arrangement- and mobility-relevant proteins using Western blotting assay [20,54]. Protein samples were obtained using RIPA buffer (Solarbio, Beijing, China) with protease/phosphatase inhibitor (Roche, Basel, Switzerland). Protein concentrations of the samples were measured using bicinchoninic acid assay (Solarbio, Beijing, China). After gel electrophoresis, target proteins would be transferred to the PVDF membranes and sealed using Tris-buffered saline plus 5% non-fat dry milk at 37 °C for 30 min. Then, the membranes were incubated using the primary antibodies of RhoA (Sigma, Burlington, MA, USA, 1:1000), ROCK1 (Sigma, Burlington, MA, USA, 1:1000) and GAPDH (Sangon Biotech, Shanghai, China, 1:500) at 4 °C overnight. Then, these protein bands were further incubated using corresponding secondary antibody at room temperature for 1 h. The immuno-reactive bands were displayed using supersignal west femto maximum sensitivity substrate (Thermo, Waltham, MA, USA). Finally, the densitometric analysis was performed using Quantity One Software.

### 4.8. Immunofluorescence Assay

After 12% injurious stretch of human ACL fibroblasts, ROCK1 protein expression was verified using Immunofluorescence assay at 24 h. ACL fibroblasts were washed using precooled and sterile PBS, and further cell fixation was performed using 4% paraformaldehyde for approximately 10 min. Subsequently, all the samples were permeabilized using 0.25% Triton-X100 for 15 min at RT. After cell permeation, the cells were washed using precooled PBS to remove the remaining Triton-X100. The ACL fibroblasts were sealed using PBS-tween plus 1% BSA for 1 h at RT. After membrane sealing, the ROCK1 primary antibody was used to incubate these samples overnight, shaking gently in low temperature environment. Then, the ACL fibroblasts were further incubated using Alexa Fluor 488-conjugated secondary antibody (Invitrogen; 1:1000) for 1 h at RT. Finally, ACL fibroblasts were stained using DAPI (Beyotime, Beijing, China) for 15 min, and these samples were photographed by fluorescence microcopy (Olympus, Tokyo, Japan).

### 4.9. Rabbit Partial ACL Transection Model

A total of 30 New Zealand female white rabbits were used in this animal experiment. The weight of these rabbits is approximately 2.4–2.6 kg. The experiments were performed referring to the standard formulated by Huaqiao University Animal Care and Use Committee. The rabbits were divided into normal group (normal), partial ACL transection (ACLT) group, and ACLT group plus MGF (0.1, 1 and 10 μg/mL), randomly. The ACL samples were harvested after drug administration at 2 or 4 weeks, thus ten groups (three rabbits in each group) were established. All rabbits were anesthetized through auricular vein intravenous injection referring to the standard at 30 mg/kg body weight pentobarbital sodium. A half cross-section on ACL were performed on 24 rabbits at the middle site in bilateral knee joints of the hind limbs. The width of these wounds is approximately 2 mm, and outflowed blood must be cleaned clearly using physiological saline before surgical suture. Moreover, 0.24 g penicillin should be injected into these rabbits by the intramuscular injection once a day in the following three days. Then, MGF solution was injected into the articular cavity once daily in the first week, once every three days in the second week, and once on the first day in the third or fourth week. After drug administration at 2 and 4 weeks, ACL tissues were obtained for further macro evaluation and histological analysis. The criteria for macro evaluation are shown in Table S1 [55]. The stress–strain analysis was used to verify the mechanical properties of the ACL samples harvested at week 4.

### 4.10. Hematoxylin-Eosin and Masson Staining

ACL ligaments were fixed in 4% paraformaldehyde overnight under low temperature conditions. Subsequently, samples were washed using precooled PBS, dehydrated with different concentrations of ethyl alcohol, embedded in paraffin. Then, the samples were cut into 5-μm-thick sections carefully using cryostat (Leica, Wetzlar, Germany). Tissue sections were dewaxed in followed steps: xylene (10 min), xylene (10 min), absolute ethyl alcohol (5 min), 95% ethyl alcohol (5 min), 75% ethyl alcohol (5 min), 50% ethyl alcohol (5 min), and distilled water (5 min). Then, HE and Masson staining were performed according to the manual to represent ECM synthesis.

### 4.11. Statistical Analysis

The data were tested using OriginPro 8.0 software, and represented in the manuscript as the mean ± standard deviation (SD). Statistical analysis was performed by one-way ANOVA analysis of variance. Moreover, *p* < 0.05 was set as the critical significance level.

## 5. Conclusions

In summary, MGF can weaken the negative effects induced by injurious mechanical stretch on cell area, roundness, migration, adhesion through regulating PAK1, PAK2, Cdc42, Rac1, RhoA, and ROCK1 mRNA or protein expression levels. MGF also exhibited a positive effect in rabbit partial ACLT models. MGF accelerated the whole ACL repair progression and significantly recovered its mechanical properties. This study supplied an underlying clinical treatment method for ACL ruptured patients.

## Figures and Tables

**Table 1:** List of primer sequences used for quantitative real-time PCR.

Gene	Primer Sequence	Amplicon Length	Annealing Temperature
Cdc42	F: 5′-CCATCGGAATATGTACCGACTG-3′	128 bp	60 °C
	R: 5′-CTCAGCGGTCGTAATCTGTCA-3′		
Rac1	F: 5′-ATGTCCGTGCAAAGTGGTATC-3′	249 bp	60 °C
	R: 5′-CTCGGATCGCTTCGTCAAACA-3′		
RhoA	F: 5′- GATTGGCGCTTTTGGGTACAT-3′	85 bp	60 °C
	R: 5′-AGCAGCTCTCGTAGCCATTTC-3′		
ROCK1	F: 5′-AAGTGAGGTTAGGGCGAAATG-3′	219 bp	60 °C
	R: 5′-AAGGTAGTTGATTGCCAACGAA-3′		
PAK1	F: 5′-CAACTCGGGACGTGGCTAC-3′	81 bp	60 °C
	R: 5′-CAGTATTCCGGGTCAAAGCAT-3′		
PAK2	F: 5′-TGAGCACACCATCCATGTTGG-3′	86 bp	60 °C
	R: 5′-AGGTCTGTAGTAATCGAGCCC-3′		
GAPDH	F: 5′-GGATTTGGTCGTATTGGG-3′	218 bp	60 °C
	R: 5′-GCTCCTGGAAGATGGTGAT-3′		

## Data Availability

All data are included in the article and supplementary materials.

## Supplementary Materials

The following supporting information can be downloaded at: https://www.mdpi.com/article/10.3390/ijms23084331/s1.
