# Supplementary material for: Mechano Growth Factor Accelerates ACL Repair and Improves Cell Mobility of Mechanically Injured Human ACL Fibroblasts by Targeting Rac1-PAK1/2 and RhoA-ROCK1 Pathways
Source: Int J Mol Sci. 2022 Apr 14;23(8):4331. doi: 10.3390/ijms23084331 (PMC9026312; doi:10.3390/ijms23084331)
Supplement: Supplementary file 1 [file ijms-23-04331-s001.zip › ijms-1660108-supplementary.pdf]

Supplementary figures and table

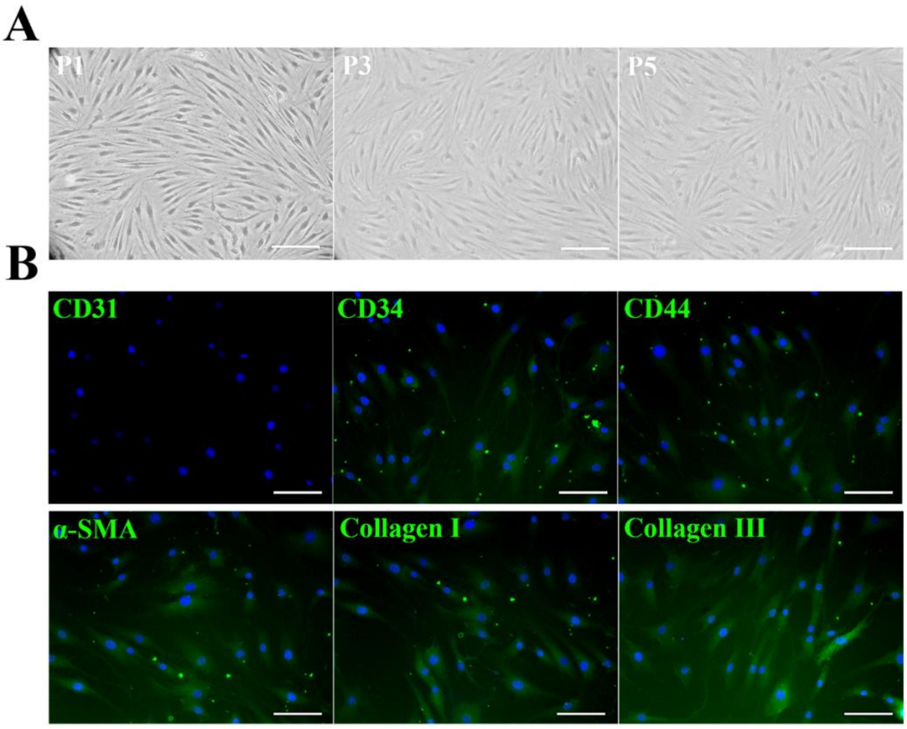

Figure S1. Cell identification of human anterior cruciate ligament fibroblasts. (A) Cell morphology of hACLFs in passage 1/3/5. (B) Expression of CD31, CD34, CD44, α-SMA and type collagen I/III were detected as cell markers through immunofluorescence staining. Scale bar= 100 μm.

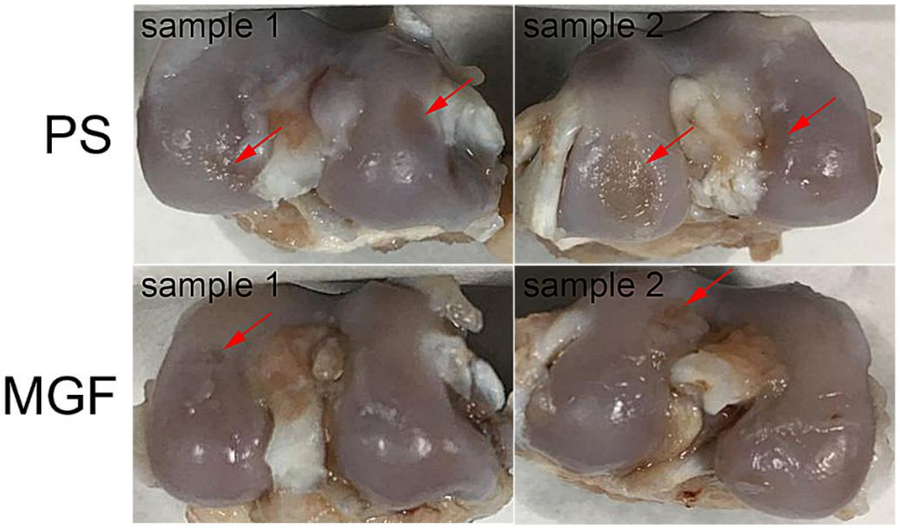

Figure S2. MGF (1 μg/ml) attenuated the progression of osteoarthritis after ACL transection surgery for 2 months in rabbits. PS: physiological saline. Red arrow: cartilage degradation.

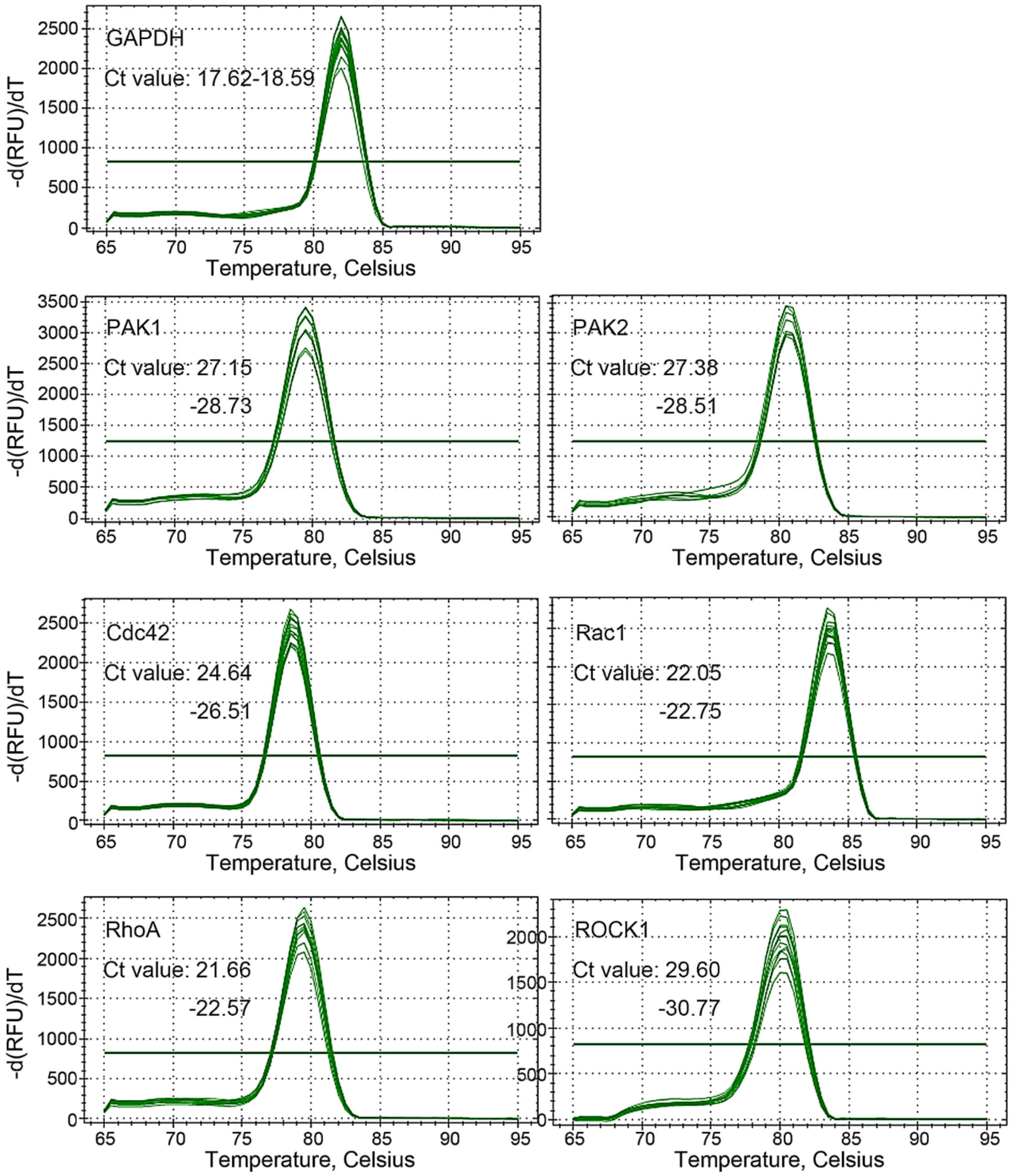

Figure S3. Melt curves and Ct values of the primes of GAPDH, PAK1, PAK2, Cdc42, Rac1, RhoA and ROCK1 in qRT-PCR analysis.

Table S1. Criteria for histological assessment.

Ligament Tissue Maturity Index (Total = 28)			
Cellularity subscore (total = 10)	Presence of inflammatory cells	Necrosis	0 points
		Polymorphonuclear cells	1 points
		No inflammatory cells	2 points
	Number of fibroblasts	None	0 points
		More than 2×normal ligament	1 points
		Less than 2×normal ligament	2 points
	Nuclear aspect ratio (NAR) of fibroblasts	No cells	0 points
		Avg NAR less than 2	1 points
		Avg NAR greater than 2	2 points
	Orientation: Long axis of nucleus parallel with normal fascicles	No cells	0 points
		Less than 30% of cells oriented	1 points
		More than 30% of cells oriented	2 points
	Arrangement of cells into columns	No cells	0 points
		Cells in columns of 2 to 3	1 points
		Cells in columns of more than 3	2 points
Collagen subscore (Total = 12)	Width of bundles	No bundles	0 points
		Width less than 50 mm	2 points
		Width greater than 50 mm	4 points
	Bundle orientation	No orientation	0 points
		Presence of bundles perpendicular to long axis of ligament	2 points
		Presence of bundles parallel to long axis of ligament	4 points
	Crimp	None present	0 points
		Crimp length < 0.5 normal length	2 points
		Crimp with normal length present	4 points
Vascularity subscore (total = 6)	Density of blood vessels	None present	0 points
		Twice as many as normal present	1 points
		Less than twice normal present	2 points
	Orientation of vessels with long axis of ligament	No vessels oriented	0 points
		Less than 30% oriented	1 points
		More than 30% oriented	2 points
	Vessel maturity	No vessels seen	0 points
		Capillaries only present	1 points
		Arterioles present	2 points
